# Skin Deep? : A scoping literature review of the psychological impact of Acne Vulgaris on patients and the role of the Psychiatrist

**DOI:** 10.1192/bjo.2021.616

**Published:** 2021-06-18

**Authors:** Stephanie Adeyemi

**Affiliations:** East and North Hertfordshire NHS Trust

## Abstract

**Aims:**

This literature review aims to assess existing scientific literature on the psychological impact of Acne Vulgaris on adolescents and adults and the role that Psychiatrists can play in supporting these patients. The hypothesis of this literature review is that all patients with Acne Vulgaris should have their quality of life assessed in order to identify those who require additional support.

**Background:**

The link between Psychiatry and Dermatology is becoming increasingly recognised. Resources on the British Association of Dermatologists’ website are often distributed to patients by Dermatologists in order to assess the impact that a skin condition has had on a patient's life. Acne Vulgaris is a psychophysiological skin condition that impacts up to 95% of people to some extent from the ages of 11 to 30 years old. Due to its prevalence it is essential that the psychological burden of Acne Vulgaris on patients is understood.

**Method:**

Literature written since 2011 was searched identified from: PsychINFO, MEDLINE, EMBASE, CINAHL and PUBMED. The search strategy key words were: acne vulgaris, mental health, psychiatry, anxiety and depression. Arksey and O'Malley's framework was utilised to conduct a scoping literature review. Data were collated and summarized thematically.

**Result:**

A total of 72 studies were included representing over 14,000 adults and adolescents with Acne Vulgaris from the following countries: Egypt, Nigeria, Turkey, India, Lithuania, UK, USA, Iran, Pakistan and Spain.

Screening tools such as the Global Acne Grading System (GAGS), The Acne Quality of Life Scale (AQLS), the Cardiff Acne Disability Index (CADI), and the State Trait Anxiety Index STAI (Y-1) form were utilised in order to identify the impact of Acne Vulgaris on patients’ quality of life and mental health. The data clearly showed the significant psychological burden that patients with Acne Vulgaris can experience. There was a clear trend of low self-esteem, lack of self-confidence, social withdrawal, depression (ranging from 23.1% to 62% of study participants), anxiety (ranging from 38.4% to 51%) and even suicidal ideation (ranging from 12.9% to 20.1%). Literature also suggested a higher prevalence of Body Dysmorphic Disorder in patients with Acne Vulgaris which should be considered and screened for.

**Conclusion:**

This scoping literature review has highlighted the significant psychological burdensome acne patients can experience. Given the prevalence of the condition Psychiatrists do have a role in working with Dermatologists to ensure appropriate screening tools are utilised and patients are able to access appropriate support.

